# Strength, Endurance, Throwing Velocity and in-Water Jump Performance of Elite German Water Polo Players

**DOI:** 10.1515/hukin-2015-0015

**Published:** 2015-04-07

**Authors:** Christoph Zinner, Billy Sperlich, Malte Krueger, Tim Focke, Jennifer Reed, Joachim Mester

**Affiliations:** 1 Institute of Training Science and Sport Informatics, The German Research Centre of Elite Sport, German Sport University Cologne, Am Sportpark Müngersdorf, Cologne, Germany.; 2 Department of Sport Science, University of Würzburg, Judenbühlweg, Würzburg, Germany.; 3 Faculty of Health Sciences, University of Ottawa, ON, Canada, USA.

**Keywords:** team sports, strength diagnostics, jump height, anaerobic and aerobic testing

## Abstract

The purpose of this study was threefold: 1) to assess the eggbeater kick and throwing performance using a number of water polo specific tests, 2) to explore the relation between the eggbeater kick and throwing performance, and 3) to investigate the relation between the eggbeater kick in the water and strength tests performed in a controlled laboratory setting in elite water polo players. Fifteen male water polo players of the German National Team completed dynamic and isometric strength tests for muscle groups (adductor, abductor, abdominal, pectoralis) frequently used during water polo. After these laboratory strength tests, six water polo specific in-water tests were conducted. The eggbeater kick assessed leg endurance and agility, maximal throwing velocity and jump height. A 400 m test and a sprint test examined aerobic and anaerobic performance. The strongest correlation was found between jump height and arm length (p < 0.001, r = 0.89). The laboratory diagnostics of important muscles showed positive correlations with the results of the in-water tests (p < 0.05, r = 0.52–0.70). Muscular strength of the adductor, abdominal and pectoralis muscles was positively related to in-water endurance agility as assessed by the eggbeater kick (p < 0.05; r = 0.53–0.66). Findings from the current study emphasize the need to assess indices of water polo performance both in and out of the water as well as the relation among these parameters to best assess the complex profile of water polo players.

## Introduction

Water polo is a team sport characterized by high-intensity, intermittent exercise with varying energetic demands (Melchiorri et al., 2010). During match play, 50–60% of athletes’ energy is derived by aerobic, 30–35% by anaerobic and 10–15% by anaerobic-lactic pathways (Snyder 2009). Strong and well-developed alternating circular movements of the legs known as the eggbeater kick are important in resisting fatigue and optimizing aerobic endurance performance. The energy derived from anaerobic pathways is necessary for jumps, short sprints and wrestle opponents. Total swimming distance during a standard water polo match (4 × 8 min) ranges from 1500 to 1650 m (Melchiorri et al., 2010; Snyder 2009). The heart rate exceeds 150 beats per min for the majority (92%) of net playing time (Snyder 2009). As a result of these varying cardiovascular and metabolic demands, water polo players require optimal physical fitness to achieve success (Melchiorri et al., 2010; Smith 1998; Tan et al., 2009).

The eggbeater kick is a form of treading water that allows water polo players to keep afloat in an upright position while the arms are free to shoot, pass, dribble and control the ball. The player’s torso is upright while the thighs are parallel, knees bent and lower legs perpendicular to the water surface. As the left leg makes a clockwise rotation, the right leg makes a counter clockwise rotation. Water polo players are able to produce “boosts” by using the eggbeater kick to propel their hips out of the water. Game analyses have shown that more than 330 changes in direction occur during an elite water polo match in which the eggbeater kick is used (Tan et al., 2009). Activity is intermittent, with durations lasting less than 15 s (Lilley 1982; Smith 1998). Elite players must, therefore, possess a high degree of skill to perform these high-intensity eggbeater movements.

Although goal scoring is essential for winning a water polo match, a high level of throwing velocity and precision is also of crucial importance. Elite male water polo players achieve maximal throwing velocities of 58 to 88 km·h^−1^ (Darras 1998). Several investigators have noted that the eggbeater kick is the most important skill in achieving high vertical reach above the water surface to clear the opponents defence and reach high throwing velocities (Feltner and Taylor 1997; Marrin and Bampouras 2008). To our knowledge, studies evaluating the eggbeater kick and strength performance measured in laboratory conditions in elite water polo players are scarce.

The purpose of this study was threefold: 1) to assess the eggbeater kick and throwing performance using a number of water polo specific tests, 2) to explore the relation between the eggbeater kick and throwing performance, and 3) to investigate the relation between the eggbeater kick in the water and strength tests performed in a controlled laboratory setting in German elite national squad water polo players. The knowledge gained from this study may aid designing effective conditioning programs for elite water polo players.

## Material and Methods

### Participants

Members of the German elite national squad participated in this study (n=15). During an initial visit, all water polo players received detailed study information and participation requirements and all provided written informed consent. The study was approved by the German Sport University in Cologne, Germany and conducted in accordance with the Declaration of Helsinki. All participants were instructed to be adequately hydrated and refrain from consuming alcohol or caffeine for 24 hours and food for 3 hours prior to each test.

### Procedures

In brief, isometric and dynamic strength of the water polo players was measured in a controlled laboratory setting. Four hours following these measures, the participants performed six water polo specific tests in a swimming pool in a randomized order.

### Strength Diagnosis

The 5 kN force sensors (Mechatronik, Hamm, Germany) in different strength machines (Edition-Line, Gym80 International MbH, Gelsenkirchen, Germany) assessed maximal voluntary isometric and dynamic strength for the lower limb adductors and abductors as well as abdominal muscles (left and right in “twister” machine). A “pull-over” machine measured the forces produced by the pectoralis muscles (major and minor) separately for the left and right side. The athletes performed three trials per muscle with one minute of rest between each of them. The investigators verbally encouraged the athletes throughout all trials to ensure maximal effort.

For the dynamic maximal voluntary contraction the athletes moved a counter weight with a fixed percentage of the isometric maximal voluntary contraction determined by pretests. The highest maximal voluntary isometric and dynamic strength achieved was used for statistical analysis. Trials with an initial counter movement, identified by a visible drop in the signal preceding the force curve, were discarded. Maximal voluntary contraction was calculated by averaging the values of force registered during 600 ms, which included the instant of maximal peak force. For all trials, the rotating axis of the machine was adjusted to align in a perpendicular manner with the participants’ individual joint axis.

### Water polo specific testing

To measure the endurance performance of the eggbeater kick, participants carried an added weight (+12.5% of their body weight) above the water surface for as long as possible, while performing the eggbeater kick. The test stopped when either the participant’s chin or elbow dropped below the water surface.

To measure the agility endurance of the eggbeater kick, the participants positioned themselves in the middle of the two goal posts and then had to alternately touch two balls (installed in the upper corners of the two water polo goalposts) as often as possible within 60 s. All participants conducted three trials and received 70 s of rest at the pool edge in the water between each trial.

A modified jump-and-reach test performed in the pool assessed the maximal vertical jump (MVJ). Participants started in a prone position on the water surface and then tried to reach the highest possible point on a 3-m jumping platform. The highest of three jumps was measured with a high-definition video camera (Sony, Minato, Tokio) positioned on the opposite edge of the pool, and used for statistical analysis.

Maximal throwing velocity (MTV) was measured using a power shot in the water. Participants positioned themselves in the water 5 m in front of the goal line and in the middle of the goal posts. A sports radar gun (SpeedtracX, Prescott WI, USA) installed one metre behind the middle of the goal line measured the throwing velocity of each shot. The fastest of three shots was used for further analysis.

### Anaerobic performance

The test to measure anaerobic performance lasted 45 s and consisted of two parts that immediately followed one another. First, participants carried an additional weight (+12.5% of their body weight) above the water surface for 15 s. The added weight was then dropped and the participants immediately sprinted for 30 s in front crawl with a semi-tethered resistance belt (+10% of their body weight) attached at their waist. The goal of this test was to cover as much distance as possible in the 30 s sprint.

### Aerobic performance

A 400 m front crawl swim assessed aerobic performance. In contrast to typical front crawl swimming, the participants should not touch the wall at the end of the lane. Instead, they touched two buoys placed 25 m apart from each other in the middle of a 50 m pool. Therefore, they swam 16 laps (400 m) between the buoys as fast as possible to simulate a swim during a water polo match.

### Metabolic blood sample

Blood samples (20 µl) were collected from the participants’ right ear lobe once they finished the last lap of the aerobic performance test (400 m front crawl swim) as well as 1, 3 and 7 minutes after the anaerobic performance test using a capillary tube (Eppendorf, Germany). Samples were measured in duplicate for blood lactate concentration using an amperometric-enzymatic sensor (Ebio Plus, Eppendorf AG, Hamburg, Germany). The mean of the two measures was used for statistical analysis.

### Statistical Analysis

In a pre-study the technical error of measurement (%TEM) for maximal voluntary isometric and dynamic strength of the pectoralis (4.2%), adductor (4.0%), abductor (4.1%) and abdominal (4.2%) muscles was obtained from 10 water-polo players on two separate days. The TEM for all in-water tests showed acceptable coefficient of variation for the multiple-trial tests. Specifically, the within participant coefficient of variation for the endurance eggbeater, agility eggbeater, throwing velocity, jumping height, anaerobic and aerobic tests was 3%, 8%, 2%, 3%, 6% and 3%, respectively.

In our laboratory, the routinely measured coefficient of variation in repeated measures of blood lactate is 1.2% at 12 mmol·L^−1^. All data were calculated with conventional procedures and reported as means ± standard deviation (SD). An alpha value of p ≤ 0.05 was considered statistically significant. All data were analyzed using Statistica® (Version 7.0, StatSoft, Inc., Tulsa, OK, USA).

## Results

The elite water polo players were 16.0 ± 3.0 years of age (range: 14–24 yrs), 185 ± 9 cm tall, weighed 84.0 ± 12.5 kilograms and exhibited a mean arm length of 81.3 ± 4.3 cm. The mean values, standard deviations and ranges for all strength and in-water test measures are shown in Table 1.

Tables 2 and 3 show the correlation coefficients [r] and corresponding p-values for all test variables. The strongest correlation observed was between maximal jump height and arm length (p < 0.001, r = 0.89) (Table 2). For the strength variables, maximal voluntary dynamic strength (W) was significantly correlated with the other results (Table 3).

A significant positive relationship between the eggbeater tests (endurance eggbeater (EE) and endurance agility eggbeater (EaE)) and jump height (J) (EE vs. J: p = 0.01, r = 0.65; EaE vs. J: p = <0.001, r = 0.86) was observed in our elite water polo players. A moderate relationship between throwing velocity and jump height was also detected (p = 0.04, r = 0.54).

Several significant correlations were observed between strength variables and the endurance agility eggbeater test. Specifically, agility endurance of the eggbeater kick was positively correlated with the dynamic strength of all muscles except for the adductors. The throwing velocity was positively correlated with the strength of the left abdominal muscles and both pectoralis muscles.

## Discussion

To our knowledge, this is the first study to measure the dynamic and isometric strength of important muscles and muscle groups for elite water polo players. We measured the strength of the adductors and abductors of the legs, abdominal muscles and pectoralis muscles of elite water polo players. We did not observe any significant muscular imbalances between the left and right abdominal muscles or pectoralis muscles (left vs. right). Findings from the endurance agility eggbeater test showed positive correlations with nearly all muscles, except for the adductor muscles.

An important contribution of the current study is that a significant, positive relation between the eggbeater tests (endurance eggbeater and endurance agility eggbeater) and jump height was observed in our elite water polo players. In water polo, a high vertical reach is essential for a strong defense as well as an accurate and fast throwing technique. The careful screening of athletes for those with a high vertical reach may be helpful for coaches in selecting strong water polo players as well as developing appropriate training strategies to improve this skill within the maturation of youth players.

Kondrič et al. (2012), Uljević et al. (2013), and Idrizović et al. (2013) reported smaller jumping heights in their testing batteries. These smaller heights when compared to those of the current study may be explained by the younger age of the junior and youth athletes in these studies. Additionally, our elite water polo players were, in comparison to their peers, physically accelerated. We are aware that the age range of our elite water polo players is large; however, this sample and the jumping data reported herein are representative of national water polo team members.

In the two following studies, a mean vertical jump height of 68 cm for national level male water polo players (Platanou 2005) and of 62 cm for elite female water polo players (Platanou and Varamenti 2011) were reported. Similar heights (71 cm) measured with a 3D video graphic technique have also been reported in other studies (Sanders 1999). It is important to note that Platanou (2005) did not include arm length in his measure of the vertical jump height. In the current study, arm length was significantly correlated with the jump height. Not surprisingly, this finding suggests that long arms may be advantageous for athletes, allowing them to reach a fair distance above the water for blocking and throwing. To compare our findings with those of Platanou (2005), we subtracted arm length from the vertical jump height measured in the current study. This calculation revealed that our athletes reached a greater jump height of 75 cm than the athletes of Platanou (2005) and Sanders (1999). The greater vertical reach we observed may be explained by a slightly higher performance level of the athletes since all participants were members of the national team whereas only nine of the athletes in the Platanou’s (2005) study were national team members.

As goal scoring is a prerequisite for success in water polo, a high level of throwing velocity and precision is needed. Uljević et al. (2013) reported relatively inconsistent accuracy for throwing precision in water polo players. We therefore decided to measure throwing velocity in the current study. Water polo players competing in the 1997 World Championships in Athens achieved a mean throwing velocity of 73 km·h^−1^ (range: 58 – 88 km·h^−1^) (Darras 1998). The wide range in throwing velocity was likely attributed to the varying performance levels of the athletes. Athletes in the current study achieved a mean throwing velocity of 69 km·h^−1^ (range: 60.0 – 75.0 km·h^−1^). Several factors may affect one’s throwing pattern, including technical abilities and effective muscular power transmission during the kinetic chain. Our findings revealed a moderate relationship between throwing velocity and jump height, suggesting that optimal height (not maximal height) is needed in achieving maximal throwing velocity (Davis and Blanksby 1977; Elliot and Armour 1988; Feltner and Taylor 1997). The world-class water polo players competing in the 1997 World Championships in Athens are comparable in their skill level to our German elite players. In point, they achieved similar throwing velocities (Darras 1998); only our younger German players achieved lower values (data not shown). Several investigators that measured the throwing velocity of junior and youth water polo players reported comparable values to the young athletes in the current study (Idrizovic et al., 2013; Uljevic et al., 2013).

A high level of aerobic and anaerobic capacity is needed to resist fatigue and maintain concentration during water polo matches. Athletes often cover more than 1600 m during a typical water polo match (Melchiorri et al., 2010). Thus, well developed aerobic and anaerobic energy systems help to comfortably cover this distance. When accounting for the distance covered during net playing time (54 m·min^−1^) and intensity of the intervals, 25% of the distance is covered at velocities greater than 1.8 m·s^−1^ (Hollander et al., 1994; Melchiorri et al., 2010). The 400 m times of Canadian national water polo players range between 268 and 306 s (Smith 1998). This is comparable to 400 m times achieved by male competitive swimmers. A highly ranked Spanish national team showed faster times of 266 and 278 s (Rodriguez 1994), which is similar (278 s) to the top four team at the 2003 world championships (Tsekouras et al., 2005). The water polo players of Smith (1998) and Rodriguez (1994) were allowed to push off the wall after each lap. The push off may help to explain the faster times achieved by these two national teams (Canadian, Spanish) when compared to the findings of the current study (268–306 s vs. 348 s). A disadvantage in using the 400 m test to assess endurance performance is that athletes and coaches do not obtain any additional feedback from these tests other than performance time. It is therefore not possible to compare improvements in anaerobic capacity from times achieved during the 400 m swim test. To improve the usefulness of a single swim test, a procedure such as the step-test protocol which includes several steps until exhaustion (Zinner et al., 2011) could be used. In summary, our findings clearly indicate that high levels of aerobic and anaerobic performance are required during water polo matches.

Although we observed no significant relationship between leg movements (cyclical and non-cyclical) and anaerobic and/or aerobic tests, it is important to note that these variables may independently contribute to success in elite water polo.

## Conclusion

To our knowledge, this is the first study to measure the dynamic and isometric strength of important muscles and muscle groups (leg adductor, leg abductor, abdominal, pectoralis) in elite water polo players. The in-water endurance agility eggbeater test was found to be significantly related to nearly all laboratory strength tests (leg abductor, abdominal, pectoralis) and in-water tests (endurance eggbeater, endurance agility eggbeater, throwing velocities, maximal jump height, aerobic test). Except for the adductor muscles, a significant positive relation between muscular strength and at least one in-water test was detected. Many significant correlations observed among the strength, endurance and technical parameters reflect the complexity of water polo training and performance demands. The above described tests are an adequate way for coaches and athletes to diligently monitor improvements in training throughout the season or during special training periods. Altogether, findings from the current study emphasize the need to assess indices of water polo performance both inside and outside the water as well as the relation among these parameters to best assess the complex profile of a water polo player.

## Figures and Tables

**Table 1:** Means (± SD) and range of all strength related and water polo specific muscles and variables of elite German water polo players. (MVC = maximal voluntary contraction, HR = heart rate, La = lactate)

***Muscle group***	***Parameter***	***Mean ± SD***	***Range***
adductor	MVC_iso_ [N]	1745.3 ± 458.0	1094 – 2624
	rel. MVC_iso_	20.9 ± 5.0	15.2 – 30.6
	[N·kg^−1^]		
	MVC_dyn_ [W]	418.5 ± 122.0	231 – 625

abductor	MVC_iso_ [N]	1404.7 ± 419.2	726 – 2166
	rel. MVC_iso_	16.6 ± 3.6	11.0 – 24.4
	[N·kg^−1^]		
	MVC_dyn_ [W]	358.0 ± 104.7	172 – 553

abdominal (right)	MVC_iso_ [N]	2451.1 ± 382.4	1655 – 3115
	rel. MVC_iso_	29.4 ± 3.3	25.5 – 37.3
	[N·kg^−1^]		
	MVC_dyn_ [W]	559.9 ± 136.7	342 – 837

abdominal (left)	MVC_iso_ [N]	2490.6 ± 449.0	1843 – 3555
	rel. MVC_iso_	29.8 ± 3.3	24.2 – 35.9
	[N·kg^−1^]		
	MVC_dyn_ [W]	521.6 ± 151.3	258 – 771

pectoralis (right)	MVC_iso_ [N]	822.3 ± 236.0	428 – 1256
	rel. MVC_iso_	9.8 ± 2.1	5.0 – 12.3
	[N·kg^−1^]		
	MVC_dyn_ [W]	381.9 ± 102.3	234 – 616

pectoralis (left)	MVC_iso_ [N]	773.9 ± 248.1	349 – 1289
	rel. MVC_iso_	9.2 ± 2.2	4.1 – 12.6
	[N·kg^−1^]		
	MVC_dyn_ [W]	370.9 ± 92.3	236 – 566


***Test***	***Mean ± SD***	***Range***
Endurance eggbeater test [s]	23.80 ± 7.13	14.8 – 38.0
Number of jumps during 3-min endurance agility eggbeater test [n]	75.87 ± 11.26	64.0 – 102.0
Throwing velocity [km·h^−1^]	68.53 ± 4.82	60.0 – 75.0
Jump height [cm]	155.87 ± 8.61	138.0 – 174.0
Anaerobic test		
- length [m]	16.27 ± 3.77	11.40 – 23.45
- peak blood lactate concentration [mmol·L^−1^]	11.78 ± 1.68	9.7 – 15.0
- peak heart rate [bpm]	171.60 ± 10.36	150 – 185
Aerobic test		
- duration [min]	5:48 ± 0:25	5:14 – 6:27
- peak blood lactate concentration [mmol·L^−1^]	7.86 ± 2.26	4.4 – 11.6

**Table 2:** Correlations and p-values between all test variables performed in-water

		*EE*	*EaE*	*TV*	*MJH*	*Anaerobic test*	*Aerobi c test*	*Arm length*
Endurance eggbeater [s]	r		*0.54*	0.39	*0.65*	0.50	0.35	0.52
	p	1	0.04^*^	0.15	0.01^*^	0.06	0.20	0.49
Number of jumps during 3-min endurance agility eggbeater test [n]	r			*0.54*	*0.86*	0.23	*0.57*	*0.83*
	p		1	0.04^*^	<0.001^*^	0.40	0.03^*^	<0.001^*^
Throwing velocities [km·h^−1^]	r				*0.54*	0.78	0.20	0.45
	p			1	0.04^*^	0.08	0.47	0.91
Maximal Jump height [cm]	r					0.51	0.35	0.89
	p				1	0.19	0.20	<0.001^*^
Anaerobic test [m]	r						0.16	0.04
	p					1	0.34	0.90
Aerobic test [min]	r							0.43
	p						1	0.11
Arm length [cm]	r							
	p							1

EE = Endurance eggbeater, EaE = Endurance agility eggbeater, TV = throwing velocity, MJH = maximal jump height

**Table 3:** Correlations between strength related variables obtained from laboratory testing and in-water specific test variables in elite German water polo players

		*strength related variables*					

		*adductor*	*abductor*	*abdominal (right)*	*abdominal (left)*	*pectoralis (right)*	*pectoralis (left)*
Endurance eggbeater [s]	r	0.04	0.02	0.13	0.13	0.16	0.17
	p	0.90	0.94	0.64	0.66	0.56	0.54
Number of jumps during 3-min endurance agility eggbeater test [n]	r	0.44	*0.53*	*0.56*	*0.61*	*0.66*	*0.58*
	p	0.10	0.04^*^	0.03^*^	0.02^*^	0.01^*^	0.02^*^
Throwing velocities [km·h^−1^]	r	0.31	0.57	0.37	*0.67*	*0.52*	*0.70*
	p	0.26	0.03	0.17	0.01^*^	0.047^*^	0.004^*^
Maximal Jump height [cm]	r	0.27	0.38	0.28	*0.49*	*0.61*	0.46
	p	0.40	0.16	0.31	0.06^*^	0.02^*^	0.08
Anaerobic test [m]	r	−0.05	−0.12	−0.28	−0.31	−0.10	−0.11
	p	0.86	0.66	0.31	0.26	0.73	0.69
Aerobic test [min]	r	−*0.53*	−0.45	−0.48	−0.33	−0.47	−0.36
	p	0.04^*^	0.09	0.07	0.23	0.08	0.18
